# Neuroprotective Effect of D-Pinitol Against MPTP-Induced Parkinsonism in C57BL/6J Mice

**DOI:** 10.3390/antiox15010059

**Published:** 2026-01-01

**Authors:** María del Carmen Juárez-Vázquez, María Leonor González-Rivera, Ramsés Maximiliano Ramírez-Martínez, Athzirys Alejandra Melecio-Hernández, Juan C. Jiménez-Cruz, Gabriela Josefina López-González, Juan Carlos Barragán-Gálvez, Juan Ramón Zapata-Morales, Judit A. Aviña-Verduzco, Angel Josabad Alonso-Castro

**Affiliations:** 1 Department of Pharmacy, Natural and Exact Sciences Division, University of Guanajuato (UG), Guanajuato 36000, Guanajuato, Mexico; carmen.juarez@ugto.mx (M.d.C.J.-V.); leonor.gonzalez@ugto.mx (M.L.G.-R.); rm.ramirez.martinez@ugto.mx (R.M.R.-M.); aa.meleciohernandez@ugto.mx (A.A.M.-H.); gj.lopezgonzalez@ugto.mx (G.J.L.-G.); jcbarragang@gmail.com (J.C.B.-G.); juan.zapata@ugto.mx (J.R.Z.-M.); 2 Secretariat of Science, Humanities, Technology, and Innovation (SEHCITI)-Institute of Chemical Biological Research, Michoacan University of San Nicolas de Hidalgo, Morelia 58030, Michoacán, Mexico; juan.carlos.jimenez@umich.mx; 3 Institute of Chemical Biological Research, Michoacan University of San Nicolas de Hidalgo, Av. Francisco J. Múgica, s/n, Edificio B-1, Ciudad Universitaria, Morelia 58030, Michoacán, Mexico; jaavina@umich.mx

**Keywords:** Parkinson’s disease, cyclic polyol, MPTP, neuroprotective, apoptosis

## Abstract

Parkinson’s disease is the second most common neurodegenerative disease in the world. Natural products can offer a possible option of neuroprotective agents for preventing neurodegenerative diseases. D-Pinitol is a cyclic polyol with anxiolytic and antidepressant effects in acute assays. This work aimed to evaluate the effects of D-Pinitol (10, 50, and 100 mg/kg p.o.) in a chronic reserpine-induced depression model (19 days), using the forced swimming and tail suspension tests in female Balb/c mice, and the neuroprotective effects in an MPTP-induced Parkinsonism model (30 days) in male C57bL/6 mice, using behavioral tests such as wire grip, rotarod, catalepsy, and others. D-Pinitol showed low antidepressant-like effects in the reserpine-induced chronic depression model, compared to amitriptyline (25 mg/kg p.o.). D-Pinitol protected MPTP-treated mice from motor impairment with similar effects to those shown by L-Dopa (25 mg/kg p.o.) as evaluated in different behavioral tests. The inhibition of oxidative stress markers, increase in dopamine levels, and avoidance of apoptosis in neuronal cells were the mechanisms by which D-Pinitol protects MPTP-treated mice from motor impairment.

## 1. Introduction

Depressive disorder is a mental disorder involving prolonged sadness, loss of pleasure in living, a lack of interest in activities, and thoughts about dying. Approximately 5.7% of adults (4.6% of men and 6.9% of women) suffer from depression [1]. Depression is associated with monoamine dysfunction, and the pharmacological treatment is based on inhibiting monoamines such as serotonin and noradrenaline reuptake and inhibiting monoamine oxidase, the enzyme responsible for degrading monoamines. However, many of these antidepressant drugs lack effectiveness in some patients due to genetic and endocrine factors and abnormalities in neurotransmitter production [2]. In addition, antidepressant drugs might induce adverse reactions, such as dizziness, sexual dysfunction, insomnia, and motor coordination impairment, and many patients stop taking their pharmacological treatment [2].

**Figure 1:**
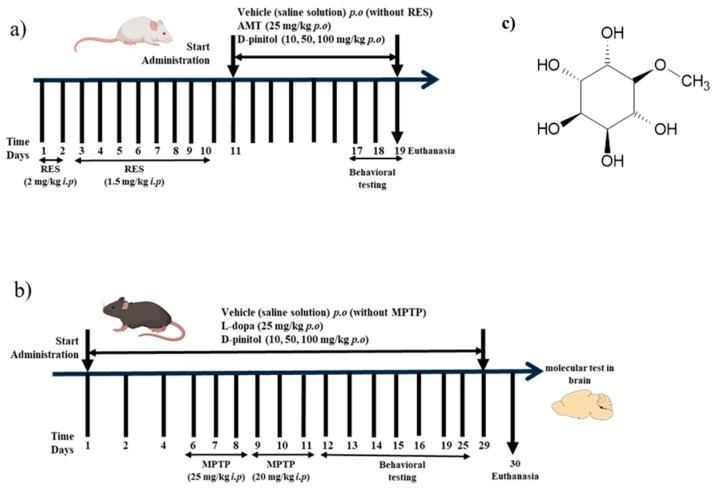
Experimental design of the (**a**) reserpine (RES)-induced chronic depression model and (**b**) MPTP-induced Parkinsonism in mice. (**c**) Chemical structure of D-pinitol.

Neurodegenerative diseases, such as Parkinson’s disease (PD), are projected to become a leading cause of death worldwide by 2040. According to the WHO, Parkinson’s disease is the second most common neurodegenerative disease in the world, and it is estimated that the number of people (25.2 million) suffering from this disease will increase by 2050 because of aging and population growth worldwide [3]. The clinical manifestations of the disease include deterioration of motor function, characterized by slow movements, tremors, and disturbances in gait and balance resulting from the loss of dopaminergic neurons in the substantia nigra of the midbrain [4,5]. In Parkinson’s disease (PD), the neuronal degeneration is linked to the dysregulation of B-cell lymphoma (Bcl-2) family proteins and to the increase in inflammatory mediators such as tumor necrosis factor-α (TNF-α), interleukin-1β (IL-1β), interleukin-6 (IL-6), and COX-2 [6,7]. The neuroinflammation and degeneration of monoaminergic neurotransmitter systems may be one of the causes of the development of a non-motor manifestation, such as moderate to severe depression. It is estimated that it may affect between 40 and 50% of patients with PD and affect the decision to take symptomatic therapy for motor impairment in PD [8]. Currently, the treatment of neurodegenerative diseases provides temporary relief of symptoms but fails to stop the progression of the disease, and often, due to its progression, higher doses are required. The most used pharmacological treatment is levodopa, a dopamine precursor; however, continuous administration of the drug can cause dyskinesia in patients [9]. D-Pinitol (3-O-Methyl-D-chiro-inositol) belongs to the family of methylated inositol (cyclitols) (Figure 1c). It is a main component of the Leguminosae family, but it has also been identified in other families such as *Pinaceae*, *Asteraceae*, *Caryophyllaceae*, *Zygophyllaceae*, *Cupressaceae*, *Aristolochiaceae*, and *Sapindaceae* [10]. D-Pinitol has been described as having diverse pharmacological properties, such as antioxidant, antidiabetic, anti-inflammatory, and cytotoxic against cancer cells [11,12,13]. The neuropharmacological properties of D-Pinitol have been investigated in different behavioral tests in murine models [14]. In the exploratory cylinder model, D-Pinitol demonstrated anxiolytic-like effects (ED_50_ = 70 mg/kg p.o.) and antidepressant-like activity (ED_50_ = 26 mg/kg p.o.) in the forced swimming test, both showing similar effects to those of the reference drugs clonazepam (1.5 mg/kg p.o.) and fluoxetine (20 mg/kg p.o.), respectively [14]. The participation of the GABAergic and noradrenergic systems might be involved in the anxiolytic-like and antidepressant-like effects, respectively, shown by D-Pinitol [14]. The chronic oral administration of D-Pinitol (200 mg/kg/day for 18 weeks) showed an improvement in cognition, improved the circulation of procognitive hormones (such as ghrelin and leptin), reduced the levels of hippocampal Aβ and hyperphosphorylated tau, and increased the expression of the insulin-degrading enzyme in 5 × FAD mice with symptoms similar to Alzheimer’s disease (AD) [15]. This study aims to evaluate the antidepressant activity of D-pinitol in a reserpine (RES)-induced depression model in female Balb/c mice (Figure 1a), as well as its neuroprotective effects in a model of Parkinson-like symptoms induced by a neurotoxin in male C57BL/6 mice (Figure 1b) and its molecular mechanisms in these neuropharmacological effects.

## 2. Materials and Methods

### 2.1. Reagents and Drugs

D-Pinitol (95% purity), L-Dopa-(phenyl-d3), DMSO, and reserpine (RES) were obtained from Sigma-Aldrich (St. Louis, MO, USA), whereas the neurotoxin 1-Methyl-4-Phenyl-1,2,3,6-Tetrahydropyridine (MPTP) was from Toronto Research Chemicals Inc. (North York, ON, Canada). Amitriptyline (AMT) was from Cayman Chemical (Ann Arbor, MI, USA).

### 2.2. Animals

Adult male C57BL/6 mice (8–10 weeks, weighing 28 ± 2 g) or female BALB/c mice (25–35 g) were obtained from the University of Guanajuato’s bioterium and housed in cages at 20 ± 5 °C and a relative humidity of 55% with access to LabDiet 5001^®^ food and water ad libitum in an environment with a 12:12 h light-dark cycle. The University of Guanajuato’s Research Ethics Committee reviewed and approved the experimental protocol with registration number CEPIUG-P40-2021, on 26 August 2021. The experiments were carried out in accordance with NOM-062-ZOO-1999. The animals were acclimatized for a period of 2 weeks before starting each experimental protocol. This work reduced as much as possible all stress, pain, discomfort, and suffering in mice. The PASS v.6 program (NCSS, Kaysville, UT, USA) calculated the control and experimental sample sizes. A beta value of 0.2, an alpha risk of 0.05, and a two-tailed test type were considered. The selection of D-pinitol doses (10–100 mg/kg p.o.) for this study was based on the previous work evaluating the antidepressant-like effects in acute assays [14].

### 2.3. Reserpine-Induced Chronic Depression in Mice

Female BALB/c mice were randomly assigned to 6 groups, each with 10 animals, and administered for 10 days. Mice of similar age were randomly assigned to each group. No randomization sequence was used. The RES group consisted of mice that received RES for 10 days (2 mg/kg i.p. for 2 days and 1.5 mg/kg i.p. for 8 days) [16]. Animals treated with RES i.p. received AMT (25 mg/kg p.o.) or D-Pinitol at doses of 10, 50, and 100 mg/kg orally for 10 days starting on day 11, and the vehicle group consisted of oral administration of saline solutions (untreated mice). D-Pinitol, RES, and AMT were solubilized in saline solutions. Behavioral tests were performed on days 11, 17, 18, and 19. The experimental procedure is described in Figure 1a. After 19 days, the animals were euthanized. This protocol was previously standardized in our laboratory, and all animals developed depression-like symptoms. No mice were excluded from this study.

### 2.4. MPTP-Induced Parkinsonism in Mice

D-Pinitol and L-Dopa were solubilized, each, in saline solution and kept for 5 min in a Branson 180 ultrasonic bath and stored at 4 °C until use. MPTP was dissolved in DMSO and used immediately. Animals were randomly assigned into 7 groups with 8 animals in each group. Mice of similar age were randomly assigned to each group. No randomization sequence was used. The vehicle group consisted of the oral administration for 29 days with saline solution (mice without any treatment). The MPTP group consisted of mice that were intraperitoneally administered with MPTP for 6 days, receiving 25 mg/kg the first 3 days and 20 mg/kg for the next 3 days [17]. Other mice received L-Dopa (25 mg/kg p.o.) for 29 days and MPTP i.p. for 6 days, as described in the MPTP group, or D-Pinitol (10, 50, and 100 mg/kg p.o.) daily for 29 days p.o. and MPTP i.p. for 6 days, as described in the MPTP group. This protocol was previously standardized in our laboratory, and all animals developed Parkinsonism-like symptoms. No mice were excluded from this study. Behavioral tests were performed on days 12, 13, 14, 15, and 16. The experimental procedure is described in Figure 1b. Body weight was recorded at the beginning and the end of the experiment. After 30 days, the animals were euthanized to obtain liver, kidney, and spleen. The relative weight of the organs was determined using the formula (organ weight/animal weight) × 100. The brains were stored in an ultra-low temperature freezer at −70 °C.

### 2.5. Behavioral Tests

#### 2.5.1. Motor Coordination

The test utilized a rotating rod apparatus (Panlab Harvard apparatus, Holliston, MA, USA) set to a constant speed of 4 revolutions per min. Mice underwent training for four consecutive days before the test to remain on the rotarod device. On the day of the motor coordination test, the animals were placed on the rotarod, and the latency to fall was recorded with a maximum cut-off time of 240 s, evaluated at 0, 60, and 120 min [14]. The test was measured on days 12 and 19 of the MPTP-induced Parkinsonism and on day 18 of the reserpine-induced chronic depression models.

#### 2.5.2. Catalepsy

Each mouse was lifted by its tail and allowed to place its forepaws on a horizontal wooden bar that was 4 cm high. The time that the mouse maintained the position with both forepaws extended was recorded in seconds (s). The end of the CAT time was considered when both forepaws were removed from the bar or if the animal moved its head exploratorily [18]. Three independent measures were taken, allowing the animals to rest. The test was measured on day 13 of the MPTP-induced Parkinsonism model.

#### 2.5.3. Paw Grip Endurance or Wire Grip

Each mouse was placed on the wire lid of a conventional rodent cage. The lid was gently shaken to induce gripping, and the mouse was turned upside down (180°) over a landing area filled with soft material. The latency (s) until the mouse released both hindlimbs and landed was recorded, with a cut-off time set at 120 s [19]. This test was measured on day 14 of the MPTP-induced Parkinsonism model.

#### 2.5.4. Exploratory Cylinder

Mice were individually housed in open acrylic cylinders (45 cm high, 20 cm in diameter, with a 3 mm wall). The number of hindpaw lifts and the number of times each mouse touched the cylinder wall with both forelimbs over a 5-min period were recorded [14]. The test was measured on day 15 of the MPTP-induced Parkinsonism model and on day 17 of the reserpine-induced chronic depression model.

#### 2.5.5. Tail Suspension

Adhesive tape was placed 1 cm from the tip of the mice’s tail, and they were suspended 50 cm from the floor on the edge of the table. Immobility time (s) was assessed for 6 min [20]. The test was measured on day 25 of the MPTP-induced Parkinsonism model and on day 17 of the reserpine-induced chronic depression model.

#### 2.5.6. Forced Swimming

Animals were placed individually in clear plastic cylinders measuring approximately 25 cm in height by 10 cm in diameter, containing water (temperature approximately 25 °C) at a depth of 15 cm. The test lasted 6 min. The final 4 min were recorded, including the total immobility time for each animal [20]. The test was measured on day 19 of the reserpine-induced chronic depression model.

### 2.6. Obtention of Protein Extracts from Brain Tissue (MPTP-Induced Parkinsonism Model)

The tissue homogenization, the protein quantification, and the Western blot analysis were carried out as previously described [21]. Briefly, tissue homogenization was performed in the BeadBug equipment (Benchmark Scientific, Inc., Sayreville, NJ, USA) with three cycles of 45 s at 3700 rpm and 2-min rests on ice. The homogenates were centrifuged at 12,500 rpm for 15 min to recover the supernatant. Approximately 190 µL of Pierce Coomassie Plus Assay Reagent (Bradford, Waltham, MA, USA) was added to each sample. Absorbances were determined in a ChroMate^®^ microplate (Awareness Technology, Inc., Palm City, FL, USA) reader at a wavelength of 630 nm. A BSA standard curve and coefficient of determination (R^2^) were obtained. Sample concentrations were obtained by interpolation in the equation of the straight line. Protein extracts were separated using polyacrylamide gels (10 or 15%). Electrophoresis was performed at 90 V for 2 h under denaturing conditions with SDS (sodium dodecyl sulfate). Subsequently, the proteins separated on the polyacrylamide gel were electrophoresed (20 V/1 h) to PVDF (polyvinylidene fluoride) membranes with a 0.20 µm pore size using the MiniBlot module (Thermo Fisher, Waltham, MA, USA). Once the electroblotting was complete, Ponceau red was added. The transferred membranes were blocked with 10% *v*/*v* milk in 1× Tris-buffered saline (TBS) and incubated with antibodies (Bcl-2, Bax, and β-Actin) from Santa Cruz Biotechnologies (Santa Cruz, CA, USA) overnight at 4 °C. The proteins were visualized using the iBright Imaging system (Thermo Fisher).

### 2.7. Obtention of Brain Tissue Homogenates for Dopamine Determination (MPTP-Induced Parkinsonism Model)

Six hundred microliters of lysis solution (prepared in distilled water and containing 150 mM sodium chloride, 50 mM Tris-HCl at pH 8.0, 1% Triton, and supplemented with protease and phosphatase inhibitors) was added to 100 mg of brain tissue and placed at −20 °C for 10 min. The tissue was then lysed using a LABGIC^®^ homogenizer (Beijing Labgic Technology Co., Ltd., Beijing, China) at 18,000 rpm for 40 s. The supernatant was separated by centrifuging the lysate at 12,500 rpm for 5 min (PRISM Microcentrifuge Labnet, Labnet International, Inc., Woodbridge, NJ, USA), performing a total of 3 cycles with the sample maintained at −20 °C for 2 min between each cycle. The supernatant was aliquoted and kept at –20 °C until analysis.

### 2.8. Estimation of Dopamine Levels Brain Tissue Homogenates (MPTP-Induced Parkinsonism Model)

The methodology was carried out according to the procedure indicated in the commercial ELISA test of the Enzo^®^ (ENZ-KIT188, Enzo Biochem, Inc., Farmingdale, NY, USA). Briefly, the procedure began with washing the wells with the 1X washing solution twice. Subsequently, 50 μL of the different concentrations of the dopamine calibration curve (1.56, 3.13, 6.25, 12.5, 25, 50, and 100 ng/mL) or homogenates (the sample dilution was 1:2) were added, and immediately, 50 μL of the biotin detection antibody (1:100 dilution) was added. The wells were covered and placed at a temperature of 37 °C for 45 min. The solution was discarded, and the wells were washed 3 times with the 1X wash solution (duration of each wash = 2 min, volume of 300 μL). Next, 100 μL of the streptavidin-HRP conjugate (dilution 1:100) was added to the wells and incubated at 37 °C for 30 min. Then, the solution was discarded, and the wells were washed 5 times with the 1X wash solution, followed by adding 90 μL of the 3,3′,5,5′-tetramethylbenzidine substrate and its incubation at 37 °C for 20 min. Finally, 50 μL of the stop solution was added to the wells, and the samples were read using the 450 nm filter without a differentiating filter (less than 20 min) using an ELISA reader. The equation obtained was an exponential decay; the natural logarithm of the dopamine concentrations was calculated to obtain a linear equation that would allow interpolation of the test samples. The results of the test samples were expressed in units of ng of dopamine/mg of total protein.

### 2.9. Total Protein Quantification Using the Commercial Bicinchoninic Acid Assay

The bovine serum albumin calibration curve was prepared in the lysis solution described in Section 2.6. The albumin concentrations were as follows: 1000, 2500, 4000, 5500, 7000, 8500, and 10,000 μg/mL. To measure the total protein, 5 μL of the calibration curve standards or homogenates (1:2 dilution) were added to 100 μL of the working reagent (50 parts of reagent A with 1 part of reagent B). The mixture was then shaken and left to sit at 25 °C for 5 min. The 492 nm filter without a differentiating filter was used to read samples without a differentiating filter on the Chromate-4300 ELISA reader (Awareness Technology Inc., Palm City, FL, USA).

### 2.10. Obtention of Brain Tissue Homogenates for Glutathione Determination

Five hundred microliters of 5% 5-sulfosalicylic acid solution were added to 100 mg of brain tissue and placed at −20 °C for 2 min, followed by vortexing for 30 s. The tissue was then lysed using a LABGIC^®^ brand homogenizer at 18,000 rpm for 20 s. The homogenate was placed at −20 °C for 10 min. The supernatant was separated by centrifugation of the lysate at 10,000× *g* for 10 min (PRISM Microcentrifuge Labnet). The supernatant was separated into aliquots and kept at −20 °C until analysis.

### 2.11. Glutathione Estimation in Brain Homogenates

The methodology followed the instructions provided in the commercial glutathione test (CS0260) from SIGMA ALDRICH^®^ (Merck KGaA, Darmstadt, Germany). The procedure began by adding 10 μL of either the glutathione calibration curve concentrations (3.125, 6.25, 12.5, 25, and 50 µM) or the homogenates (with a sample dilution of 1:4), followed immediately by the addition of 150 μL of the working reagent, which consists of glutathione reductase enzyme solution (6 U/mL) and DTNB stock solution (1.5 mg/mL) prepared in 1× assay buffer. The resulting mixture was then incubated at 25 °C for 5 min. After this time, 50 µL of NADPH at a concentration of 0.16 mg/mL was added, and the samples were read every min for 5 min using the ELISA reader equipment with a 405 nm filter and no differentiating filter. The results were expressed as µM of reduced glutathione/mg of tissue.

### 2.12. Estimation of Myeloperoxidase (MPO) Activity and Nitric Oxide (NO) Levels in Brain Homogenates

The assays for estimating the MPO activity and the NO levels were performed following the procedures described in detail by Barragan-Galvez et al. [22]. Myeloperoxidase activity and nitric oxide were determined using an ELISA reader with 630 nm and 492 nm primary filters without a differentiating filter, respectively. Results were reported as mOD/mg of protein for MPO activity, and the nitrite concentration was determined by extrapolation from the sodium nitrite standard curve (7–100 μM).

### 2.13. Computational Study

Conformational searches for D-Pinitol were performed using the Spartan’20 software (Wavefunction, Inc., Irvine, CA, USA, 2020. Q-CHEM). Subsequently, the conformer with the highest population contribution was selected, and its geometry was optimized by using the Gaussian16 software [23], employing B3LYP/6-311+G(d,p) as the level of theory. Molecular docking simulations were conducted using structures obtained from the Protein Data Bank. The structure containing an acylsulfonamide ligand (PDB ID: 2O22) [24] was implemented for Bcl-2, whereas PDB ID: 1F16, which lacks a reference drug, was selected for Bax [25]. The structure corresponding to subform C, containing the inhibitor 7-((3-(1-methyl-1H-pyrazol-3-yl) benzyl) oxy)-1H-[1–3] triazolo [4,5-b] pyridin-5-amine (PDB ID: 5QJ2) [26], was employed for myeloperoxidase. Additional targeted molecular docking was carried out using the monoamine oxidase B (MAO-B) structure (PDB ID: 2V5Z) [27] and the dopamine transporter (DAT) (PDB ID: 8Y2D) [28], two key proteins involved in the metabolism of MPTP and the transport of the active form MPP+ [29]. Water molecules and additional ligands were removed from each protein subunit for protein preparation. Polar hydrogens were added, and partial Gasteiger charges were assigned. Molecular docking was performed using a population of 5000 individuals, with 100 runs for directed docking and 1000 runs for blind docking, using the default parameters in AutoDock 4.2 [30]. The docking methods were validated by redocking, where proteins containing cocrystallized molecules (2O22 and 1F16) showed RMSD values < 2 Å. The visualization of the results and molecular association positions was performed using Discovery Studio (Biovia, D.S. Discovery Studio Visualizer. 2019. San Diego, CA, USA) and UCSF Chimera (version 1.17.6) [31].

Molecular dynamics simulations evaluated the evolution and stability of the optimal molecular docking positions by using the NAMD software (version 3.0.1) [32]. The CHARMM-GUI [33] and Solution Builder [34] modules, employing the CHARMM36 force field, performed the complex parameterization [35]. The generated cubic cell was adjusted to a pH of 7.4 and solvated with the TIP3P water model under periodic conditions. NaCl ions at 0.15 M were added to keep the system neutral. Long-range electrostatic interactions were modeled using the Ewald Particle Mesh (PME) method [36]. Periodic conditions were applied during the simulation process to avoid boundary effects. Initially, minimization was performed using the conjugate gradient algorithm. Subsequently, the system was heated from 0 to 310 K for 500 ps, maintaining the temperature for another 500 ps under the NVT array, using the Langevin thermostat [37]. The system was then equilibrated for 5 ns in the NPT array (1 atm and 310 K). Finally, the production phase was carried out for 100 ns, integrated using 2 fs time steps. The molecular dynamics results were analyzed using root mean square deviation (RMSD), and hydrogen bonding interactions were calculated using VMD software (version 1.9.3) [38].

### 2.14. Statistical Tests

All data were expressed as the mean ± standard error of the mean (SEM) and analyzed using a one-way or two-way analysis of variance followed by Tukey’s and Dunnett’s multiple comparisons test. The statistical software used was GraphPad Prism (version 9.5.0). The probability value of *p* < 0.05 was considered a statistical difference.

## 3. Results

### 3.1. D-Pinitol Slightly Improves Depression-like Behavior and Motor Activity in a Reserpine-Induced Chronic Depression Model

**Figure 2:**
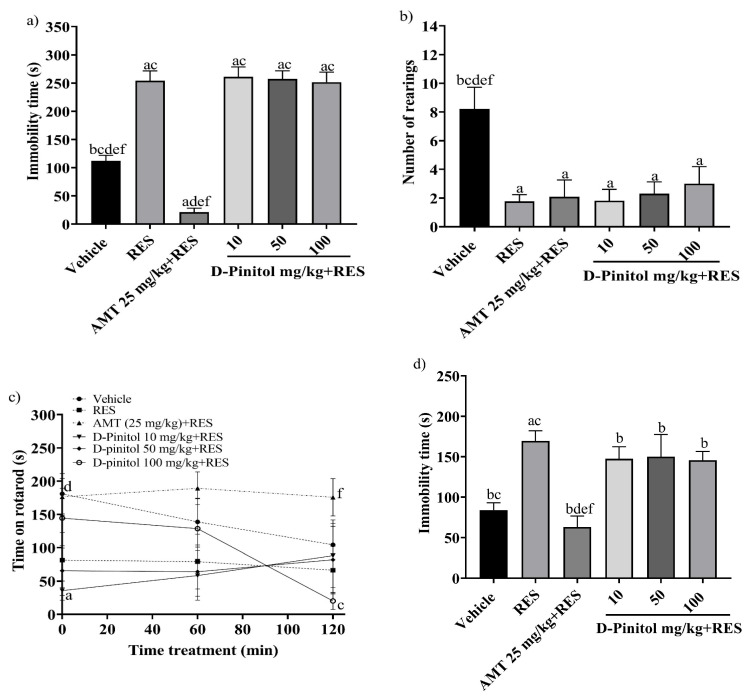
The following behavioral tests were performed in the reserpine-induced chronic model: (**a**) the tail suspension test on day 17, (**b**) the exploratory cylinder test on day 17, (**c**) the motor coordination on day 18, and (**d**) the forced swimming test on day 19. Data are expressed as mean ± SEM. One-way ANOVA followed by Tukey’s post hoc test (*p* < 0.05) was performed on a vs. Vehicle; b vs. RES; c vs. AMT (25 mg/kg) + RES; d vs. D-Pinitol (10 mg/kg) + RES; e vs. D-Pinitol (50 mg/kg) + RES; and f vs. D-Pinitol (100 mg/kg) + RES (*n* = 7).

The tail suspension test was assessed on day 17 in the reserpine-induced chronic depression model. The RES group exhibited a significantly (*p* < 0.0001) higher immobility time (254.2 s) compared to the vehicle group (112.4 s) (Figure 2a). The groups treated with RES+D-Pinitol at doses of 10, 50, and 100 mg/kg had immobility times of 261.2 s, 257.2 s, and 251.9 s, respectively. These times did not significantly differ (*p* < 0.0001) from the RES group’s time of 254.2 s. In contrast, the AMT 25 mg/kg + RES group demonstrated a significantly lower time of 21.33 s, serving as the reference drug (Figure 2a).

The exploratory cylinder test was conducted on day 17. The RES group showed an average of 1.76 rearings, whereas the AMT 25 mg/kg + RES group recorded 2.09 rearings. The D-Pinitol + RES groups at doses of 10, 50, and 100 mg/kg exhibited 1.81, 2.30, and 3.0 rearings. The D-Pinitol+RES groups showed a significant (*p* < 0.05) increase in the number of rearings compared to the vehicle group, which showed 8.22 rearings (Figure 2b).

The motor activity of the animals was determined on day 18. At 0, 60, and 120 min, the RES group decreased the time spent on the rotarod to 81.63 s, 79.29 s, and 66.43 s, and the vehicle group showed mobility times of 181.0 s, 138.8 s, and 104.3 s, respectively. Only the group treated with AMT 25 mg/kg + RES improved the mobility time of the animals during times 0, 60, and 20 min with values of 176.3 s, 189.3 s, and 175.8 s. The groups treated with D-Pinitol 10 and 50 mg/kg + RES at times 0 to 120 min maintained the mobility of the animals in ranges of 35 to 88 s, and at time 60 min the D-Pinitol 100 mg/kg + RES group showed an increase in mobility with 128.6 s and a decrease in its motor capacity (20.20 s) at 120 min (Figure 2c).

The forced swimming test was evaluated on day 19. The RES group increased the immobility time (169.5 s) compared (*p* < 0.05) to the vehicle group (84 s) (Figure 2d). The groups treated with D-Pinitol at doses of 10, 50, and 100 mg/kg + RES showed slight decreases in immobility time with 147.5, 150.0, and 145.6 s, respectively, compared to the RES group (169.5 s); however, only the AMT 25 mg/kg + RES group exhibited a significant decrease in the immobility time of the animals compared (*p* < 0.05) to the vehicle group (Figure 2d).

D-Pinitol showed low antidepressant-like activity in the reserpine-induced chronic depression model, compared to the reference drug AMT (amitriptyline). Therefore, no further experiments regarding the molecular mechanism of action of the antidepressant-like activity in chronic assays were performed.

### 3.2. D-Pinitol Improves Locomotor Activity in Mice with MPTP-Induced Parkinsonism

**Figure 3:**
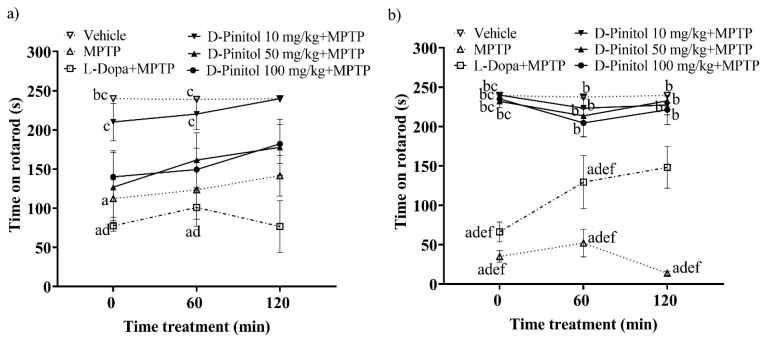
Motor coordination was assessed in an experimental model of MPTP-induced Parkinsonism at two points: (**a**) day 12 and (**b**) day 19. Data are expressed as mean ± SEM. Two-way ANOVA followed by Tukey’s post hoc test (*p* < 0.05) was performed on a vs. Vehicle; b vs. MPTP; c vs. L-Dopa (25 mg/kg); d vs. D-Pinitol (10 mg/kg) + MPTP; e vs. D-Pinitol (50 mg/kg) + MPTP; f vs. D-Pinitol (100 mg/kg) + MPTP; n = 7.

On day 12 of the MPTP-induced Parkinsonism model in C57BL/6 mice, the group treated solely with MPTP showed a significant decrease in mobility on the rotarod, recording times of 112.3 s and 123.5 s at 0 and 60 min, respectively. In contrast, the vehicle group (mice without any pharmacological treatment) maintained mobility times of 240 s and 238.9 s, respectively, on the rotarod (Figure 3a). Treatment with D-Pinitol at 10 mg/kg + MPTP improved mobility, yielding times of 210.2 s (time 0) and 220.3 s (time 60 min), which were comparable to those of the vehicle group. Mice receiving D-Pinitol at doses of 50 and 100 mg/kg + MPTP exhibited slight improvements in mobility, with times of 126.9 and 140.1 s, respectively, at 0, and 161.6 and 149.4 s, respectively, at time 60 min. The L-Dopa group experienced a more substantial reduction (*p* < 0.05) in mobility time compared to the MPTP group, recording 77.63 s at 0 min and a slight recovery to 101.0 s at 60 min (Figure 3a).

On day 19 of the experimental model, animals treated with D-Pinitol at 10, 50, and 100 mg/kg + MPTP showed values of 240.0, 232.0, and 235.1 s at time 0, values of 223.3, 213.6, and 204.6 s at 60 min, and values of 227.4, 232.7, and 221.3 s at 120 min, observing a mobility behavior similar to the vehicle group with values of 239.4, 237.4, and 239.4 s. The L-Dopa 25 mg/kg + MPTP group showed a slight increase in mobility with 66.23 s at 0 min, 129.4 s (60 min), and 148.2 s (120 min), compared (*p* < 0.05) to the MPTP group with values of 35.04, 52.09, and 13.75 s, respectively (Figure 3b).

### 3.3. D-Pinitol Protects Mice from Motor Impairment Due to MPTP-Induced Parkinsonism

**Figure 4:**
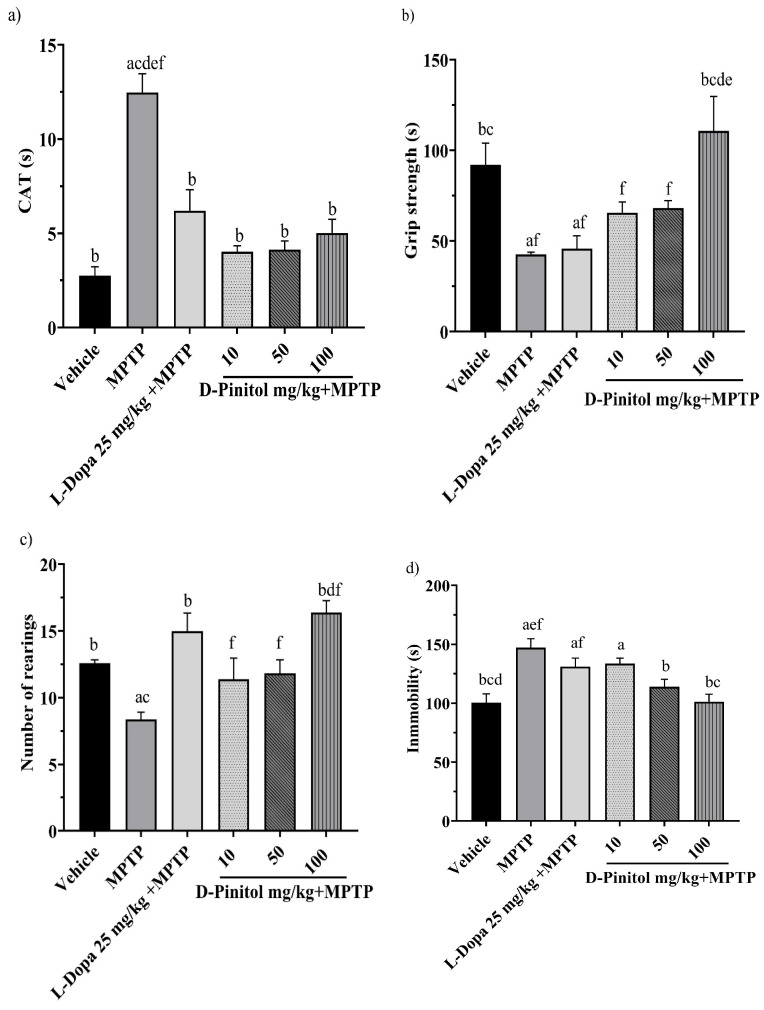
Effects of D-Pinitol on motor impairment in mice treated with MPTP. The following behavioral tests were conducted: (**a**) catalepsy test on day 13, (**b**) grip test on day 14, (**c**) exploratory cylinder test on day 15, and (**d**) tail suspension test on day 25. The data are presented as the mean ± SEM. Statistical analysis included one-way ANOVA followed by Tukey’s post hoc test (*p* < 0.05), comparing a vs. Vehicle; b vs. MPTP; c vs. L-Dopa (25 mg/kg); d vs. D-Pinitol (10 mg/kg) + MPTP; e vs. D-Pinitol (50 mg/kg) + MPTP; f vs. D-Pinitol (100 mg/kg) + MPTP; n = 7.

On day 13 of the MPTP-induced Parkinsonism model, the groups treated with MPTP + D-Pinitol (10, 50, and 100 mg/kg p.o.) and MPTP + L-Dopa (25 mg/kg p.o.) exhibited a shorter catalepsy time with 4.023, 4.139, 5.023, and 6.20 s, respectively, with values close to the vehicle group with 2.757 s. On the other hand, the MPTP group showed a catalepsy time of 12.48 s (Figure 4a).

On day 14 of the experiment, only the MPTP + D-Pinitol (100 mg/kg p.o.) group improved the grip strength time (110.9 s), compared (*p* < 0.05) to the MPTP group (42.68 s), with similar activity to that shown by the vehicle group (91.92 s) (Figure 4b).

The exploratory cylinder test was determined on day 15. It was observed that the number of rearings shown by mice from the MPTP + D-Pinitol (100 mg/kg) (16.40 rearings) or L-Dopa (25 mg/kg) (15 rearings) groups was comparable to the exploratory behavior of the vehicle group (12.60 rearings) (Figure 4c). Mice from the MPTP + D-Pinitol (10 and 50 mg/kg) groups showed an increase (*p* < 0.05) in the number of rearings (11.40 and 11.83 rearings), compared to the MPTP group (8.37 rearings) (Figure 4c).

On day 25, the forced tail suspension test was assessed. Only the MPTP + D-Pinitol (100 mg/kg p.o.) significantly (*p* < 0.05) decreased the immobility time (101.3 s) compared to the MPTP group (147.1 s), showing activity levels equivalent to those of the vehicle group (100.5 s) (Figure 4d).

### 3.4. Relative Organ Weight in the MPTP-Induced Parkinsonism

**Figure 5:**
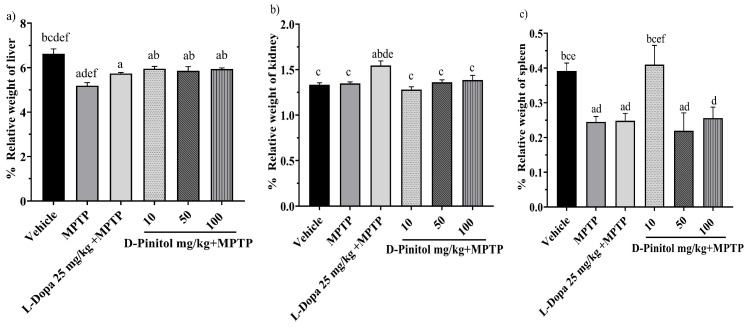
Percentage of relative weight of (**a**) liver, (**b**) kidney, and (**c**) spleen in male C57BL/6 mice with experimental MPTP-induced Parkinson’s disease on day 30. The data are presented as the mean ± SEM. Statistical analysis included one-way ANOVA followed by Tukey’s post hoc test (*p* < 0.05) comparing a vs. Vehicle; b vs. MPTP; c vs. L-Dopa (25 mg/kg); d vs. D-Pinitol (10 mg/kg) + MPTP; e vs. D-Pinitol (50 mg/kg) + MPTP; f vs. D-Pinitol (100 mg/kg) + MPTP; n = 7.

The relative liver weight decreased (*p* < 0.05) in mice in the MPTP and L-Dopa (25 mg/kg) groups by 5.19% and 5.74%, respectively, compared to the vehicle group, which showed 6.62% of the weight. In addition, D-Pinitol at 10, 50, and 100 mg/kg showed no alteration in the relative weight of the liver (Figure 5a).

The relative weight of the kidneys of the groups treated with MPTP + D-Pinitol (10, 50, and 100 mg/kg) showed a % weight of 1.28, 1.36, and 1.38, which was equivalent to the vehicle group with 1.33%. However, only the L-Dopa 25 mg/kg increased its organ weight by 1.54% (Figure 5b).

Animals treated with MPTP experienced a decrease (*p* < 0.05) in spleen weight by 0.24%. In comparison, the groups treated with L-Dopa at 25 mg/kg and D-Pinitol at doses of 50 and 100 mg/kg had organ weights of 0.24%, 0.21%, and 0.25%, respectively, while the vehicle group showed an organ weight of 0.39%. Mice from the MPTP + D-Pinitol (10 mg/kg) group showed a relative weight comparable to the vehicle group at 0.40% (Figure 5c).

### 3.5. D-Pinitol Prevents Neuronal Death in the MPTP-Induced Parkinsonism Model

**Figure 6:**
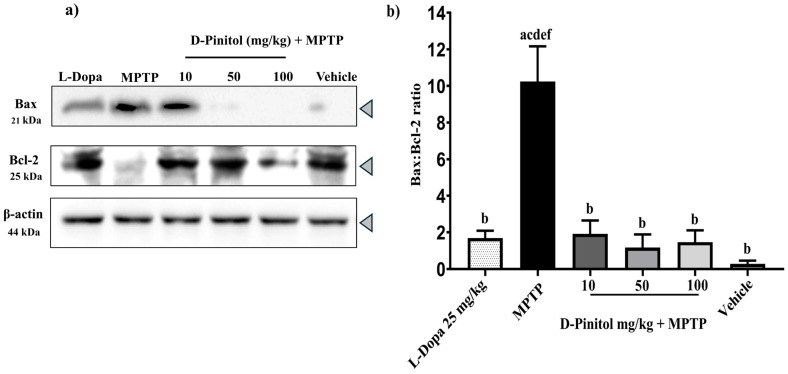
Effects of D-Pinitol on the expression of the anti-apoptotic protein Bcl-2 in the brains of MPTP-treated mice. (**a**) Western blot analysis of Bax/Bcl-2 in brain tissue from male C57BL/6 mice with experimental MPTP-induced Parkinson’s disease and (**b**) densitometric analysis. The data are presented as the mean ± SEM. Statistical analysis included one-way ANOVA followed by Tukey’s post hoc test (*p* < 0.05) comparing a vs. Vehicle; b vs. MPTP; c vs. L-Dopa (25 mg/kg); d vs. D-Pinitol (10 mg/kg) + MPTP; e vs. D-Pinitol (50 mg/kg) + MPTP; f vs. D-Pinitol (100 mg/kg) + MPTP.

The MPTP group showed a decrease in the anti-apoptotic protein Bcl-2 expression but an increase in the expression of the pro-apoptotic protein Bax, whereas the vehicle group showed an increase in Bcl-2 expression and a decrease in Bax expression (Figure 6a). Samples from the MPTP + D-Pinitol and MPTP + L-Dopa increased (*p* < 0.05) the Bcl-2 expression but decreased (*p* < 0.05) the expression of Bax (Figure 6a).

The Bax/Bcl-2 ratio of the vehicle showed minimal cell death, whereas the MPTP group showed a high proportion of neuronal death (Figure 6b). The MPTP + D-Pinitol and MPTP + L-Dopa decreased the Bax/Bcl-2 ratio with comparable values to the vehicle group (Figure 6b).

### 3.6. D-Pinitol Restores the Brain Dopamine Levels in MPTP-Treated Mice

**Figure 7:**
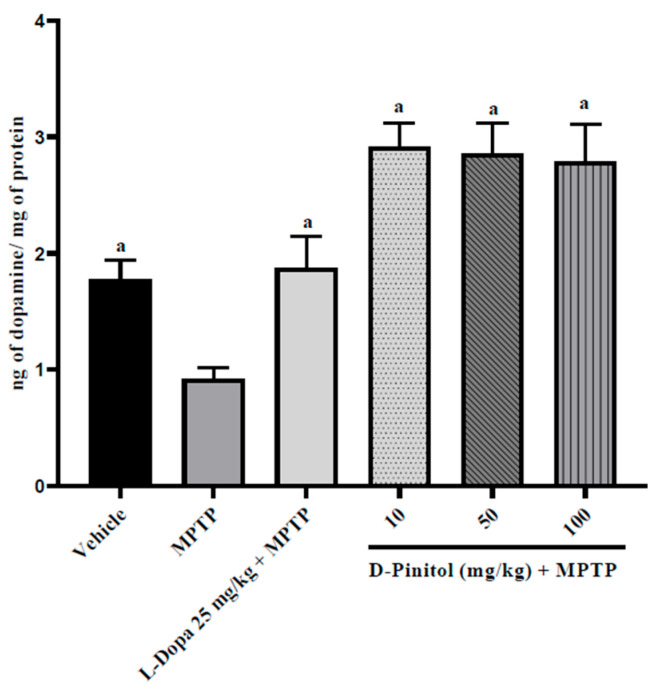
D-Pinitol restores the brain dopamine levels in MPTP-treated mice. The data are presented as the mean ± SEM. One-way ANOVA followed by Dunnett’s post hoc test (*p* < 0.05) comparing a vs. MPTP. n = 7.

D-Pinitol (10–100 mg/kg p.o.) significantly (*p* < 0.05) increased brain dopamine levels (2.03 to 2.16 times more than the group with only MPTP) in MPTP-treated mice in a dose-independent way, exhibiting higher activity than L-Dopa (25 mg/kg p.o.) + MPTP (1.04 times more than the group with only MPTP) (Figure 7). The MPTP group showed a decrease in the levels of dopamine (48.18% lower than the vehicle group), whereas the L-Dopa + MPTP group presented the same dopamine levels as the vehicle group (Figure 7).

### 3.7. D-Pinitol Increased Brain Glutathione Levels and Decreased Myeloperoxidase (MPO) Activity and Nitric Oxide (NO) Levels in MPTP-Treated Mice

**Figure 8:**
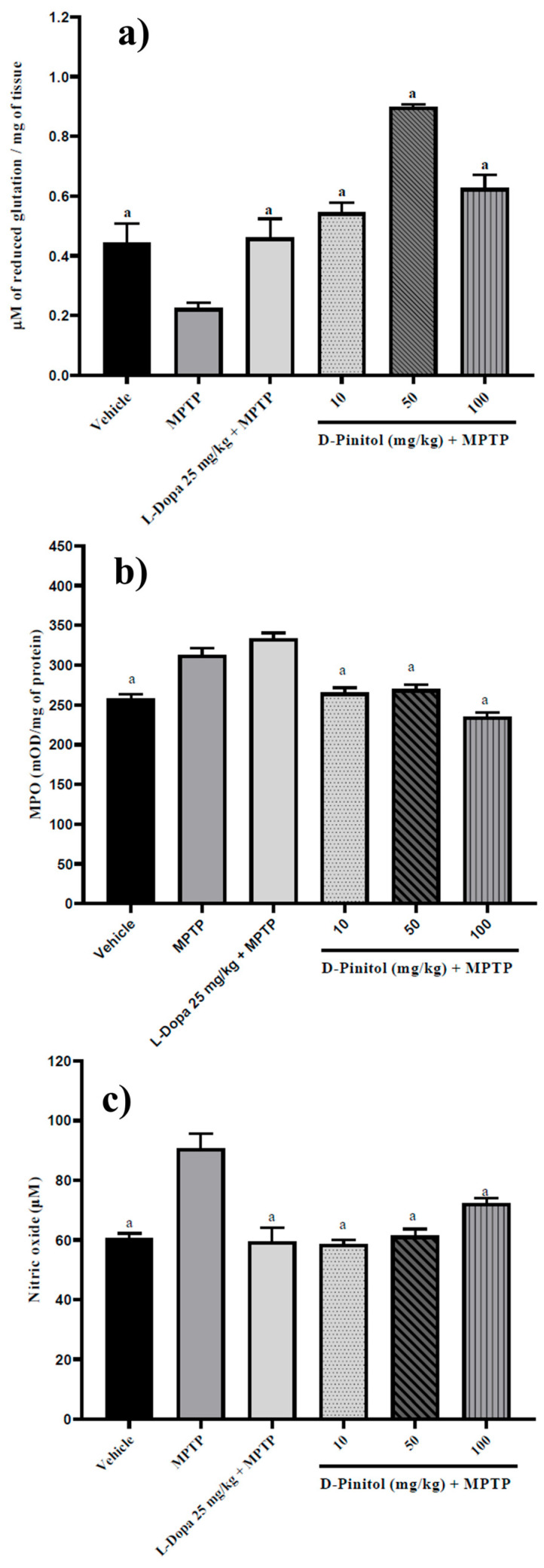
D-Pinitol increases (**a**) glutathione levels but decreases (**b**) MPO and (**c**) NO levels in brain samples from MPTP-treated mice. The data are presented as the mean ± SEM. One-way ANOVA followed by Dunnett’s post hoc test (*p* < 0.05) comparing a vs. MPTP. n = 7.

Similarly, D-Pinitol (10–100 mg/kg p.o.) significantly increased (*p* < 0.05) brain glutathione levels (1.42 to 2.99 times with respect to the MPTP group) in MPTP-treated mice in a dose-independent way. D-Pinitol (50 mg/kg p.o.) showed higher activity than L-Dopa (25 mg/kg p.o.) + MPTP (Figure 8). The MPTP group showed a decrease in the glutathione levels, whereas the L-Dopa + MPTP group restored the glutathione levels compared to the vehicle group (Figure 8a).

The levels of MPO (21% more than the vehicle group) and NO (49.61% more than the vehicle group) increased in mice treated with MPTP. On the contrary, D-Pinitol + MPTP restored the levels of MPO and NO compared to the vehicle group (Figure 8b,c). L-Dopa (25 mg/kg p.o.) showed no effects on MPO levels but restored the levels of NO compared to the vehicle group (Figure 8b,c).

### 3.8. In Silico Study

**Figure 9:**
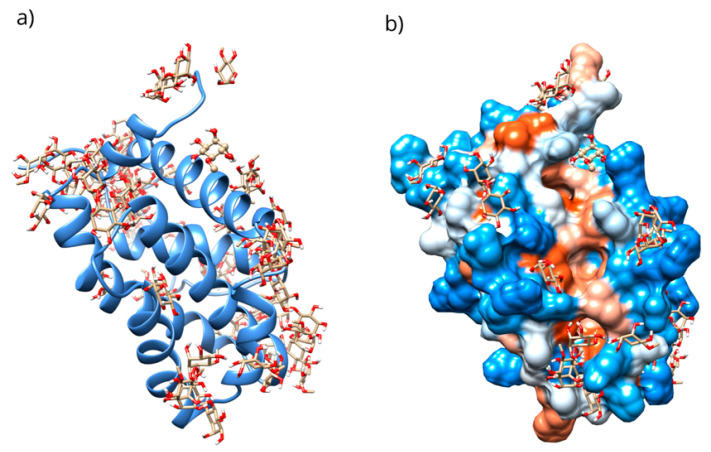
(**a**) Distribution of the conformations obtained through blind molecular docking. (**b**) The location of the selected conformation within the hydrophobic groove in the D-Pinitol-Bcl-2 complex.

The interaction of D-Pinitol with Bax and Bcl-2 was assessed using an in silico study. The results obtained, considering the complete protein and the 1000 lowest-energy conformations, showed affinity energies in the range of −5.95 to −4.04 kcal/mol (Figure 9).

**Figure 10:**
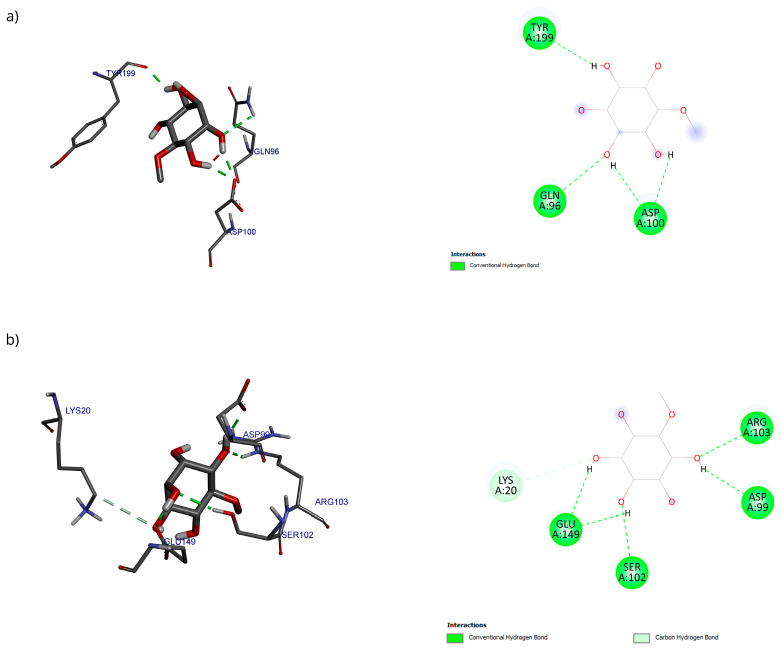
Two-dimensional and 3D interactions of D-Pinitol with Bcl-2 obtained by (**a**) blind docking and (**b**) directed docking.

Only 0.3% of the conformations were in the vicinity of the hydrophobic groove, also called the BH3 pocket (Figure 10). The best conformation obtained by blind molecular docking had an affinity energy of −4.92 kcal/mol and established hydrogen bond interactions with the residues Gln99, Asp100, and Tyr199 in Bcl-2 (Figure 10a). The directed molecular docking, centered on the region where the reference drug is co-crystallized, yielded an affinity energy of −4.93 kcal/mol for its best pose. This conformation was located below the α3 helix and below the hydrophobic groove (Figure 10).

**Figure 11:**
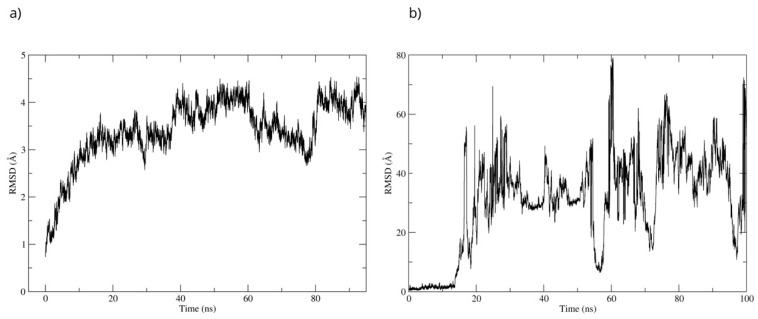
RMSD of (**a**) the Cα skeleton of Bcl-2 and (**b**) the ligand aligned to Bcl-2.

The stability of the Bcl-2–D-Pinitol complex was analyzed by using molecular dynamics simulation. The complex remained stable between 20 and 40 ns and then exhibited fluctuations, as observed with respect to the root mean square deviation (RMSD) of the protein’s Cα backbone (Figure 11a). The SE ligand’s stability was analyzed through the RMSD of the ligand aligned to the protein. The system showed stability during the first 17 ns of the simulation. Then, D-Pinitol detached from the protein-ligand complex’s binding site (Figure 11b).

**Figure 12:**
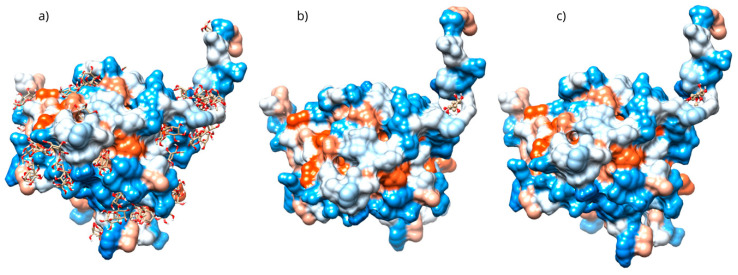
(**a**) Grouping of molecules obtained in blind docking, (**b**) best pose obtained in blind docking, (**c**) best pose obtained in directed docking with the D-Pinitol-Bax complex.

The stability of the D-Pinitol-Bax complex is supported by the fact that this interaction was adopted by 30.6% of the 1000 conformations analyzed (Figure 12a). Both experiments yielded very similar results regarding the poses in which D-Pinitol coupled to Bax (Figure 12b,c). Blind coupling showed a pose with an affinity energy of −6.62 kcal/mol, while directed coupling showed a value of −6.02 kcal/mol.

**Figure 13:**
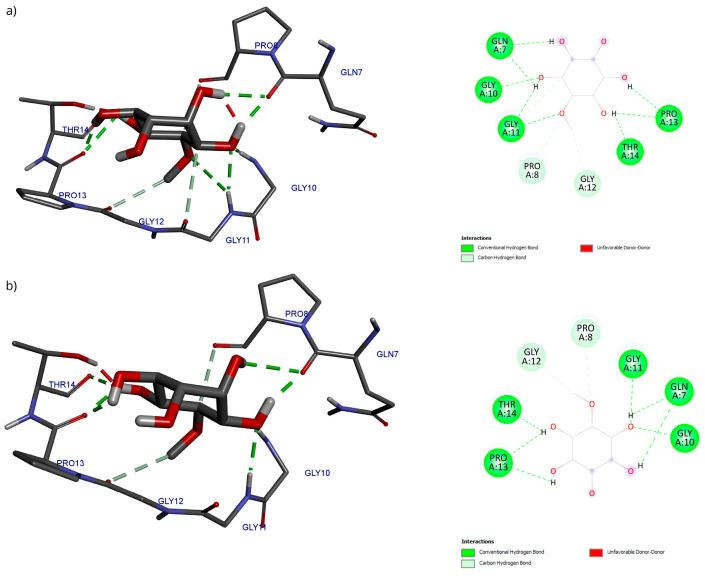
Two-dimensional and 3D interactions of D-Pinitol with Bax obtained by (**a**) blind docking and (**b**) directed docking.

Regarding the analysis of interactions in the D-Pinitol-Bax complex, a high similarity between the obtained poses can be observed in the hydrogen bonding pattern (Figure 13). In both couplings, a broad network of interactions was identified with residues Gln7, Gly10, Gly11, Pro13, and Thr14, all belonging to the N-terminal fragment of Bax (Figure 13).

**Figure 14:**
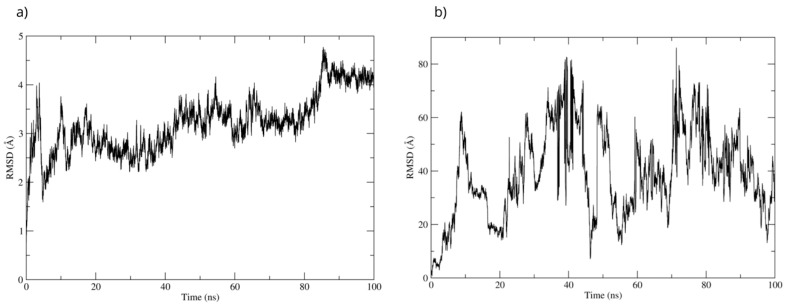
RMSD of (**a**) Bax Cα skeleton and (**b**) D-Pinitol aligned to the Bax protein.

The best pose obtained from the molecular docking was subjected to molecular dynamics for 100 ns. During the simulation, the RMSD of the Bax backbone remained stable (Figure 14a), while the Bax–D-Pinitol interaction was unstable in the first few nanoseconds, leading to D-Pinitol completely detaching from the N-terminal site of Bax (Figure 14b). This behavior suggests that the compound does not exert a relevant inhibitory or activating effect on the Bax protein.

**Figure 15:**
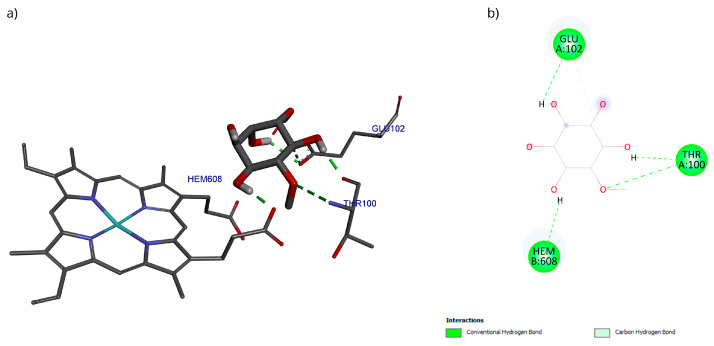
Two-dimensional and 3D interactions of D-Pinitol with MPO obtained by (**a**) blind docking and (**b**) directed docking.

The docking with the D-Pinitol and MPO complex showed that the best pose exhibited an affinity energy of −5.70 kcal/mol (reference = −9.17 kcal/mol). In this conformation, four hydrogen bonding interactions were observed: two with the Glu102 residue and one each with Thr100 and the heme prosthetic group (Hem608) (Figure 15).

**Figure 16:**
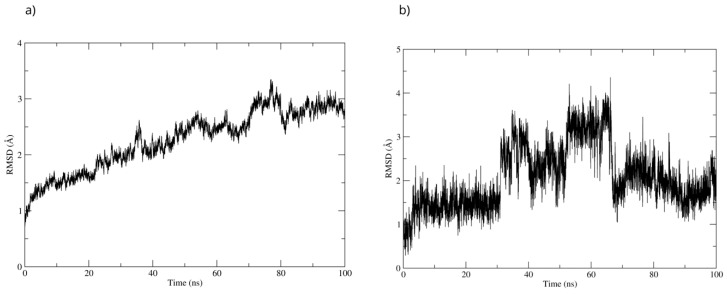
RMSD of (**a**) Bax Cα skeleton and (**b**) D-Pinitol aligned to MPO.

To evaluate the stability of the complex, a 100 ns molecular dynamics simulation was used. The complex’s high stability was observed throughout the simulation. The RMSD of the protein backbone showed that the MPO-D-Pinitol complex’s affinity gradually increased without any sudden changes (Figure 16a). This means that the protein-ligand interaction was stable. The RMSD of D-Pinitol aligned with the protein remained stable until approximately 30 ns; subsequently, it exhibited a series of conformational movements that generated significant fluctuations until 65 ns. After this point, the ligand returned to a conformation close to its initial pose and remained more stable until 100 ns. It is relevant to note that throughout the simulation, the ligand remained close to the catalytic site (Figure 16b). The observed stability is consistent with and supported by the hydrogen bonding interactions observed throughout the simulation. Among these intermolecular forces, the interactions with the Thr100 residue as an acceptor (49% occupancy), Thr100 as a donor (33%), His95 as a donor (8%), and Glu102 as a donor (11%) stand out.

**Figure 17:**
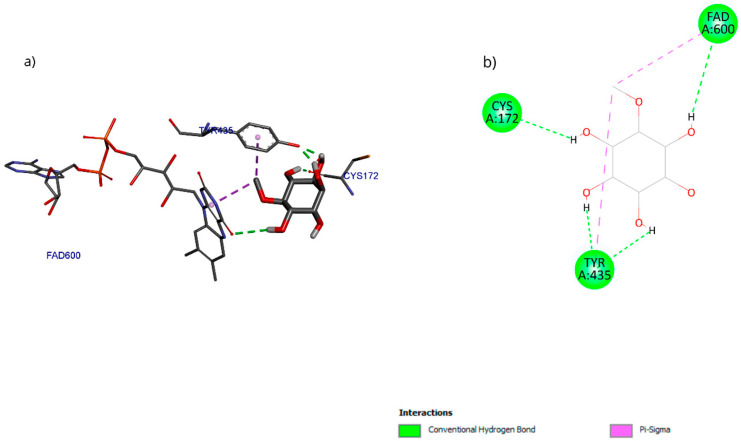
Two-dimensional and 3D interactions of D-Pinitol with MAO-B obtained by (**a**) blind docking and (**b**) directed docking.

The molecular docking results with the D-pinitol and MAO-B complex showed an affinity energy of −5.01 kcal/mol and predominant hydrogen bond interactions with residues Tyr135 and Cys172, as well as with the cofactor FAD600 (flavin adenine dinucleotide) (Figure 17).

**Figure 18:**
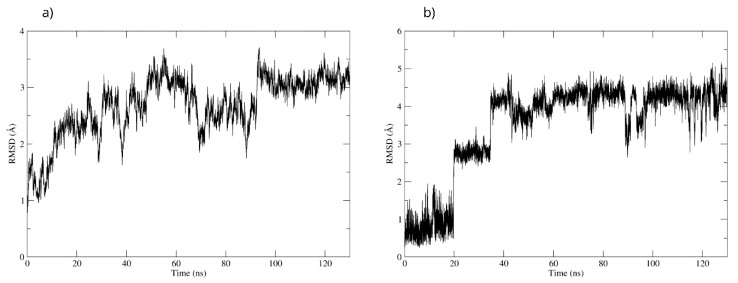
RMSD of (**a**) Bax Cα skeleton and (**b**) D-Pinitol aligned to MAO-B.

To validate the interactions observed in the molecular docking (Figure 18a), a molecular dynamics simulation was performed. The stability of the MAOB-D-pinitol complex was assessed by RMSD analysis. The results show that D-pinitol exhibited two significant fluctuations around 20 and 40 ns; however, after this period, the RMSD remained below 4 Å. Despite these initial fluctuations, the ligand remained within the catalytic site throughout the simulation (Figure 18b). Therefore, the simulation was extended to 125 ns to confirm that this stability was maintained beyond 100 ns. Hydrogen bond analysis revealed that the interactions between D-pinitol and the FAD cofactor were conserved throughout the simulation, with an occupancy of 11%. This information supports the possible inhibition of the enzyme, which could contribute to preventing the conversion of the neurotoxin MPP+. On the other hand, although the protein backbone exhibited significant fluctuations, these remained below 4 Å. However, it is important to note that after 100 ns, the simulation shows high stability (Figure 18).

**Figure 19:**
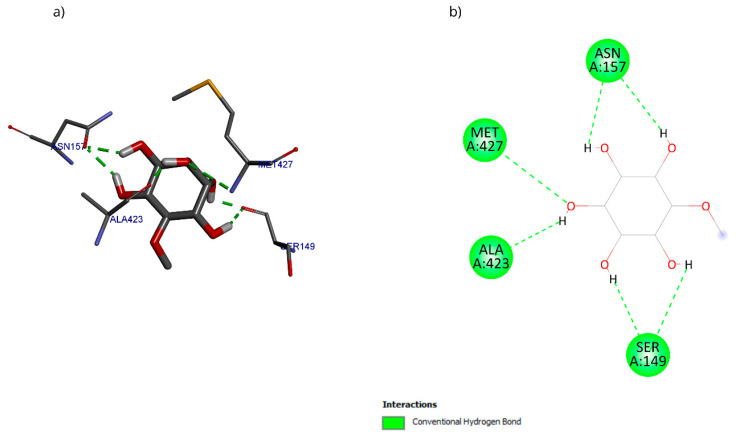
Two-dimensional and 3D interactions of D-Pinitol with DAT obtained by (**a**) blind docking and (**b**) directed docking.

The molecular docking with the D-pinitol and DAT complex showed a binding energy of −6.22 kcal/mol and allowed the identification of the interactions shown in Figure 19. D-pinitol established an extensive network of six hydrogen bonds, involving all the hydroxyl (–OH) groups of the ligand, including residues Asn157, Ser149, Ala423, and Met427.

**Figure 20:**
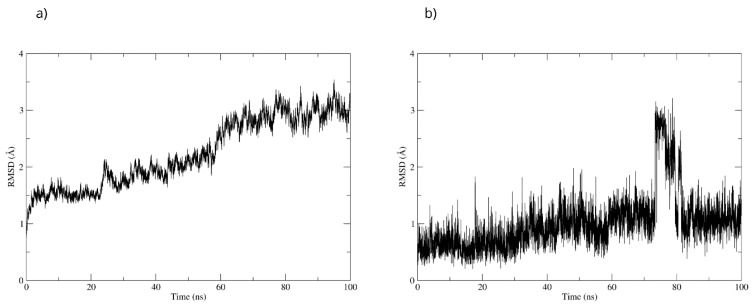
RMSD of (**a**) Bax Cα skeleton and (**b**) D-Pinitol aligned to DAT.

To validate the stability of the D-pinitol complex with DAT, a molecular dynamics simulation was performed. The RMSD analysis of the protein backbone showed overall stability, with slight fluctuations between 50 and 60 ns. Subsequently, the system exhibited remarkable stability, which was maintained until the end of the simulation (Figure 20). The RMSD of D-pinitol aligned with the protein showed high stability from the beginning of the simulation until approximately 70 ns, followed by significant fluctuation. However, from 80 ns onward, the complex stabilized again. This behavior suggests that the ligand exhibits adequate stability within the DAT inhibitor binding site. This stability can be explained by the persistent formation of seven hydrogen bonds throughout the simulation, which highlights the interactions involving residue Ser149 acting as a donor with an occupancy of 71%, the interactions with Ser422 acting as an acceptor with an occupancy of 56%, and additional interactions with Ser149 serving as an acceptor with an occupancy of 42% (Figure 20).

## 4. Discussion

D-Pinitol showed anxiolytic-like and antidepressant-like effects in acute assays [14] and modulatory effects in an Alzheimer’s disease model in mice [15]. This work evaluated the neuroprotective effects of D-Pinitol in the reserpine-induced chronic depression model and the MPTP-induced Parkinsonism model in mice.

Reserpine, an antihypertensive and psychotropic drug, blocks the secretion of 5-hydroxytryptamine in the brain, which decreases the levels of monoamines. The depletion of monoamines is one cause of the major depressive disorder [2]. Therefore, this preclinical model emulates this neurological disorder [16]. Data indicate that female mice exhibit greater sensitivity to the effect of reserpine compared to male mice. This higher sensitivity may be attributed to the fact that female mice produce more dopamine and possess a greater number of vesicles for dopamine transport, or they may have a more active form of the vesicular monoamine transporter type 2 (DAT), compared to male mice [39]. The tail suspension test and the forced swimming test involve environments that create an inescapable situation in which the mice try to escape, but they realize that the escape is impossible, and they display immobility. Animals with more immobility represent a depression-like phenotype [20]. D-Pinitol showed low antidepressant-like activities in the reserpine-induced chronic depression model; these effects were lower than those shown with the reference drug amitriptyline, a tricyclic antidepressant used for treating major depressive disorder. Therefore, no further studies with the antidepressant-like effects of D-Pinitol were performed. This information indicates that in acute assays, D-Pinitol showed antidepressant-like activity (ED_50_ = 26 mg/kg p.o.). However, D-Pinitol might be less effective in chronic depression.

Parkinson’s disease is associated with the loss of dopaminergic neurons, oxidative stress, protein aggregation, apoptotic neuronal death, and formation of intraneuronal protein inclusions (Lewy bodies) constituted by α-synuclein [17]. Patients with Parkinson’s disease experience tremor, akinesia, rigidity, and postural reflexes, and non-motor symptoms such as anxiety, depression, somnolence, and others [17].

The intraperitoneal injection of MPTP in mice induces dopaminergic neurodegeneration in C57BL/6 mice (older than 8 weeks and with more than 22 g of body weight), which shows a higher susceptibility of neuro-intoxication [18]. However, there are no differences in MPTP toxic susceptibility between male and female C57BL/6 mice [40]. Due to its lipophilicity, MPTP can pass through the blood–brain barrier and enter the central nervous system, and it is metabolized to MPP+ by the monoamine oxidase type B. MPP^+^, the active metabolite of MPTP, is selectively taken up by dopaminergic neurons and induces oxidative stress and dopaminergic neuronal death [17].

Motor impairment is one of the main symptoms of Parkinson’s disease [17]. The rotarod test evaluates the ability of mice to maintain balance and motor coordination. MPTP-treated mice present slow performance of voluntary movements and the inability to execute simultaneous actions, which are components of akinesia, a characteristic symptom of Parkinson’s disease [20]. A postural immobility state with muscular rigidity is also a characteristic of akinesia. The MPTP-treated mice showed movement impairments and increased the time of postural immobility. On the contrary, D-Pinitol (10–100 mg/kg p.o.) decreased the time of postural immobility with similar activity to that shown by L-Dopa (25 mg/kg p.o.).

The MPTP-treated mice showed a reduction in forelimb muscle strength associated with rigidity, another main symptom of Parkinson’s disease [17]. D-Pinitol (100 mg/kg p.o.) protected mice from losing muscle strength due to the MPTP administration, evaluated by the Wire Grip Test. This activity shown by D-Pinitol was comparable to the activity of L-Dopa (25 mg/kg p.o.). Other models to evaluate motor skills in MPTP-treated mice include rearing activity tests [41], such as the cylinder exploratory test. D-Pinitol (100 mg/kg p.o.) protected mice from motor impairment due to the MPTP administration, and this effect was also comparable to that shown by L-Dopa (25 mg/kg p.o.).

Depression is one of the non-motor symptoms of Parkinson’s disease [17]. MPTP induced immobility in all mice, and only D-Pinitol (100 mg/kg p.o.) decreased significantly the immobility time. This effect was comparable to that shown by the vehicle group. These findings corroborate the antidepressant effects of D-Pinitol, as shown in the acute assays [14]. However, as mentioned previously, D-Pinitol showed a slight decrease in immobility in the forced swimming test in a chronic model of induced-like depression. The MPTP model focuses more on locomotor activities.

Short- and long-term toxicological studies are necessary in drug-discovery studies to evaluate the risk of plant-derived compounds [42]. D-Pinitol also protected MPTP-induced Parkinsonism mice from body weight or organ weight loss, which are common symptoms induced by this neurotoxin. In acute assays, D-Pinitol (10–100 mg/kg p.o.) showed no locomotor impairments (evaluated with the open field test and rotarod test), and only 100 mg/kg D-Pinitol showed sedative-like effects in mice [14].

MPP^+^ reduces the expression of anti-apoptotic proteins such as Bcl-2, which leads to the release of cytochrome c and the activation of caspase 9 [19]. At the same time, MPP^+^ increases the expression of apoptotic proteins like Bax. Chronic administration of MPTP in mice results in increased lipid peroxidation in the brain, which subsequently decreases glutathione levels, elevates MPO activity and NO levels, causes dopaminergic neuronal death, and reduces dopamine levels [21]. Glutathione consists of three amino acids, L-γ-glutamyl-L-cysteinyl-glycine, and its function is to maintain the survivability of the cells. MPP^+^ also increases the concentrations of NO, with the subsequent activation of poly[ADP-ribose] polymerase 1 (PARP-1), depletion of adenosine-triphosphate and nicotinamide-adenine nucleotide, and the neuronal cell death [43]. After MPTP administration, microglia activation occurs by pro-inflammatory cytokines such as IL-1β, which trigger the production of NO and prostaglandins [44]. Then, NO reacts with oxygen or superoxide anion to induce the formation of reactive nitrogen species, catalyzed by MPO, such as nitrogen dioxide, producing the nitration of α-synuclein with the successive nigrostriatal damage [44]. The neurotoxicity of NO in the brain involves glutathione depletion, affecting neuronal proliferation and viability through DNA damage and protein oxidation, ultimately inducing apoptosis by decreasing Bcl-2 levels [44].

Patients with Parkinson’s disease experience low levels of glutathione and dopamine [45]. Myeloperoxidase (MPO) concentration and activity are increased in patients with Parkinson’s disease, and the increase in this enzyme activity is related to the progression of this neurodegenerative disease [45]. Clinical evidence indicates an increase in the levels of NO and H_2_O_2_ in neutrophils from Parkinson’s disease patients [46]. Therefore, the preclinical model of MPTP in mice corroborates clinical signs.

The inhibition of oxidative stress in the brain might be a therapeutic strategy in ameliorating the appearance and progression and decreasing symptoms of neurodegenerative diseases such as Parkinson’s disease [46]. The findings showed that D-Pinitol increased the levels of glutathione and dopamine in MPTP-treated mice. Furthermore, D-Pinitol increased an antioxidant marker (glutathione), increased the levels of dopamine, decreased the levels of pro-inflammatory markers such as nitric oxide and MPO, and protected neurons from death by increasing the expression of Bcl-2 and decreasing the expression of Bax. The inhibition of apoptosis in neuronal cells was one of the mechanisms by which D-Pinitol protects MPTP-treated mice from motor impairment. The decrease in oxidative stress markers and the increase in dopamine levels were other possible mechanisms by which D-Pinitol induces its neuroprotective effects in MPTP-treated mice. Further studies in our research group will consider other murine models, including transgenic mice, to corroborate the findings of this work.

For Bcl-2, a blind molecular docking was performed with the PDB protein (ID: 2O22) [24], even though the protein’s active site was known; this allowed for unrestricted prediction of the possible preferential binding sites of D-Pinitol. The conformations in the vicinity of the BH3 pocket correspond to the active site where drugs such as venetoclax exert their inhibitory mechanism [47]. The Tyr199 residue is in the hot spot of the Bcl-2 protein and has been associated with the stabilization energy characteristic of BH3 motif mimetics [24]. Hydrogen bonding interactions were established with residues Asp99, Ser102, and Arg103, belonging to the α3 helix [48]. The Bcl-2-D-Pinitol complex showed a low affinity and a limited association. This finding is relevant because Bcl-2 overexpression has been linked to neuroprotective properties in several studies [49]. Furthermore, the presence of Bcl-2 in murine models has been shown to decrease LPS-induced neuroinflammation in neural stem cells. Therefore, interfering with its activity could trigger uncontrolled cell death [25].

Regarding the Bax protein, both blind molecular docking and targeted molecular docking were performed (PDB ID: 1F16) [50]. The results showed a high affinity of D-Pinitol for the N-terminal region of Bax, the area that plays a crucial role in allosteric regulation and conformational stability [50]. The analysis of interactions between D-Pinitol and Bax revealed that the Pro13 residue is of great importance due to its association with the conformational changes that allow the translocation of Bax to the cytoplasmic membrane [50].

A directed molecular docking was performed using the 5QJ2 crystallographic structure [51] between D-Pinitol and MPO. The interaction between D-Pinitol and the Hem608 residue in MPO is of particular importance, since triazolopyrimidine-type compounds have been able to bind to the heme group and inhibit the oxidation of the chloride anion to hypochlorous acid [49]. In particular, the interaction of D-Pinitol with the Glu102 residue of MPO is relevant because this residue has been considered a key residue, as it has been reported to generate polar interactions with inhibitory ligands [51].

The findings showed that D-pinitol protected neurons from death induced by MPTP. However, one limitation of this study is that the role of the dopamine transporter and tyrosine hydroxylase in the dopamine secretion found is still unknown. In addition, the effects of D-pinitol on the MPTP metabolic modification, as another possible mechanism of action, will be studied. This work also evaluated the in silico interaction of D-pinitol with monoamine oxidase B, the enzyme responsible for catalyzing the conversion of MPTP to MPP+ [29]. This prediction can reflect the role of D-pinitol on the metabolism of MPTP. The affinity of D-pinitol with cofactor FAD600 is relevant since FAD600 plays an essential role in the transformation of MPTP by acting as an electron acceptor during the oxidative deamination responsible for MPP+ synthesis. In this context, the potential blocking of FAD could be associated with the inhibition of MPP+ formation [52]. The dopamine transporter (DAT) is another protein associated with Parkinson’s disease, responsible for dopamine reuptake and for transporting the dopaminergic neurotoxin MPP^+^ [53]. Inhibition of DAT could be associated with an increase in dopamine concentration in the synaptic cleft [54]. The residues Asn157, Ser149, Ala423, and Met427 in DAT have been previously associated with the recognition of DAT inhibitors [55]. D-pinitol showed an affinity for these key residues of DAT. These predictions can provide information that D-pinitol might interact with the dopamine secretion through DAT and affect the MPTP metabolism through its binding to MAO-B. However, experimental work is necessary to evaluate these possibilities.

## 5. Conclusions

D-Pinitol showed low antidepressant-like activity in a reserpine-induced chronic depression model in mice. D-Pinitol protected MPTP-treated mice from motor impairment by decreasing neuronal death, increasing the levels of dopamine, increasing the levels of the antioxidant marker (glutathione), and decreasing the levels of the pro-oxidant markers (MPO and NO). In addition, D-pinitol showed no alterations of vital organs such as the liver, kidney, and spleen in long-term administration. The in silico study corroborated that D-pinitol exerted key interactions with amino acid residues in Bax, Bad, and MPO. The in silico study also predicted the possible interaction of D-pinitol with dopamine transporter and MAO-B, which participate in the metabolism of MPTP and the transport of MPP^+^, respectively.

## Data Availability

The data presented in this study are available on request from the corresponding author.
